# Time Dilation of Quantum Clocks in a Relativistic Gravitational Potential

**DOI:** 10.3390/e27050489

**Published:** 2025-05-01

**Authors:** Tommaso Favalli, Augusto Smerzi

**Affiliations:** 1 Department of Physics, Universitá degli Studi di Trieste, Strada Costiera 11, I-34151 Trieste, Italy; 2 QSTAR, INO-CNR, and LENS, Largo Enrico Fermi 2, I-50125 Firenze, Italy; augusto.smerzi@ino.it

**Keywords:** quantum time, gravitational quantum mechanics

## Abstract

We study the dynamical evolution of two quantum clocks interacting with a relativistic gravitational potential. We find a time dilation effect for the clocks in agreement with the gravitational time dilation as obtained from the Schwarzschild solution in General Relativity. We perform our investigation via the Page and Wootters quantum-time formalism, exploring the dynamics of clocks assuming them in both a product state and a more general (entangled) state. The gravitational redshift, as emerging from our framework, is also proposed and discussed.

## 1. Introduction

The general theory of relativity predicts that spacetime outside a non-rotating, spherical mass *M* is described by the Schwarzschild metric. In spherical coordinates, this metric is given by (*c* = 1):

(1) $$ds^{2}=\left(1-\frac{R_{S}}{r}\right)dt^{2}-{\left(1-\frac{R_{S}}{r}\right)}^{-1}dr^{2}+r^{2}d\Omega^{2}$$

where dt is the coordinate time read by a far-away observer, *r* is the Schwarzschild radial coordinate, $R_{S}=2GM$ is the Schwarzschild radius, and $d\Omega^{2}$ is the metric on a unit two-sphere (see, for example, Ref. [1]).

As a consequence of (1), when considering two static clocks *A* and *B* at distances $r_{A}$ and $r_{B}$ from the origin of the field, we have:

(2) $$\frac{\tau_{B}}{\tau_{A}}={\left(1-\frac{R_{S}}{r_{B}}\right)}^{\frac{1}{2}}{\left(1-\frac{R_{S}}{r_{A}}\right)}^{-\frac{1}{2}}$$

where $\tau_{A}$ and $\tau_{B}$ are the proper times measured by the clocks *A* and *B*, respectively. Equation (2) shows that $r_{B}<r_{A}$ implies $\tau_{B}<\tau_{A}$, i.e., clock *B* is delayed with respect to *A*.

In this work, we show that a time dilation effect can emerge by considering two quantum clocks interacting with a relativistic gravitational potential [2,3]. Our clocks are described by time states belonging to the complement of a bounded Hamiltonian with discrete spectrum, as introduced in [4,5,6]. Such clocks may have discrete or continuous time values: in the first case, the complement of the Hamiltonian is described by a Hermitian operator, while in the latter case, it is described by a POVM. We address both scenarios.

In calculating the interaction between the clocks and the relativistic gravitational potential, we promote the masses of the clocks to operators using the mass–energy equivalence $m\to m+{\hat{H}}_{clock}$ [7,8,9,10]. In this framework the coupling between the clocks and the field enters as an interaction term in the global Hamiltonian, which affects the evolution of the time states. In describing such a system, we focus on the clock’s internal degrees of freedom, which are the only ones relevant to our model.

We study the dynamics of the clocks through the Page and Wootters (PaW) quantum-time formalism [11,12]. This approach to time was first proposed by D. N. Page and W. K. Wootters in 1983 and has recently gained a great deal of interest (see, for example, Refs. [6,9,13,14,15,16,17,18,19,20,21,22,23,24,25,26]), including an experimental realization [27,28]. In PaW’s theory, time is a quantum degree of freedom which belongs to an ancillary Hilbert space (which we call the *C* subspace), equipped with a suitable time observable. The dynamics of the system of interest is thus obtained with respect to the observer *C*, which we place (for convenience) at an infinite distance from the source of the field.

For a complete overview of the PaW mechanism we refer to [26]. We start by providing a brief summary of the theory in Section 2, also showing that two free clocks (throughout the whole work, we refer to free clocks as clocks not perturbed by the gravitational field) evolve synchronously with respect to the time reference *C*. In Section 3 and Section 4, we consider instead two clocks located at different distances from the origin of the gravitational field, and we show the emergence of the time dilation effect, in agreement with the Schwarzschild solution (2). We notice that this framework was already been introduced in [10] where the interaction of clocks with a Newtonian gravitational field was principally considered. Here, we focus instead on the interaction with the relativistic gravitational potential, we reinterpret the results by introducing proper times for the clocks, and we also study the evolution of entangled (and/or interacting) clocks. In Section 5, we show how the gravitational redshift can emerge in the theory. Finally, in Section 6, we give our conclusions and outlook.

## 2. Evolution of Free Clocks

We provide here a brief review of the PaW theory following the generalization proposed in [6,15,26] and show, as an example, the synchronous time evolution of two free clocks A+B with respect to the time reference *C*. The global Hamiltonian reads:

(3) $$\hat{H}={\hat{H}}_{C}+{\hat{H}}_{A}+{\hat{H}}_{B}$$

where ${\hat{H}}_{C}$, ${\hat{H}}_{A}$ and ${\hat{H}}_{B}$ are the Hamiltonians acting on *C*, *A* and *B*, respectively. The key point of the PaW formalism is to consider the global quantum system in a stationary state:

(4) $$\hat{H}|\Psi \rangle =0.$$

We notice and emphasize that the zero eigenvalue does not play a special role in identifying the state |Ψ〉. Indeed, up to an irrelevant global phase in the dynamics of A+B, the state |Ψ〉 can also be identified by imposing the constraint $\hat{H}|\Psi \rangle =\epsilon |\Psi \rangle$ with real ϵ [13].

### 2.1. The *C* Subspace

We assume that ${\hat{H}}_{C}$ has a discrete spectrum, with non-degenerate eigenstates having rational energy ratios. More precisely, we consider $d_{C}$ energy states ${|E_{i}\rangle }_{C}$ and $E_{i}$ energy levels with $i=0,1,2,\ldots ,d_{C}-1$ such that $\frac{E_{i}-E_{0}}{E_{1}-E_{0}}=\frac{\beta_{i}}{\gamma_{i}}$, where $\beta_{i}$ and $\gamma_{i}$ are integers with no common factors. We obtain (ℏ=1):

(5) $$E_{i}=E_{0}+r_{i}\frac{2\pi }{T_{C}}$$

where $T_{C}=\frac{2\pi r_{1}}{E_{1}}$, $r_{i}=r_{1}\frac{\beta_{i}}{\gamma_{i}}$ for i>1, $r_{0}=0$, and $r_{1}$ is equal to the lowest common multiple of the values of $\gamma_{i}$. We thus define the states

(6) $${|t\rangle }_{C}=\sum_{i=0}^{d_{C}-1}e^{-iE_{i}t}{|E_{i}\rangle }_{C}$$

with $t\in \left[t_{0},t_{0}+T_{C}\right]$. These states can be used for writing the resolution of the identity in the *C* subspace:

(7) $$I_{C}=\frac{1}{T_{C}}\int_{t_{0}}^{t_{0}+T_{C}}dt|t\rangle \langle t|.$$

Thanks to property (7), the time observable in *C* is represented by a POVM generated by the infinitesimal operators $\frac{1}{T_{C}}|t\rangle \langle t|dt$. This framework for the subspace *C* allows us to consider any generic Hamiltonian as a Hamiltonian for the *C* subspace. Indeed, in the case of non-rational ratios of energy levels, the resolution of the identity (7) is no longer exact but, since any real number can be approximated with arbitrary precision by a ratio between two rational numbers, the residual terms and small corrections can be arbitrarily reduced.

### 2.2. Clocks *A* and *B*

We focus now on clocks *A* and *B*. For simplicity, we take *A* and *B* to be equal, with an equally spaced energy spectrum. Furthermore, we assume $d_{A}=d_{B}=d$ and $d_{C}\gg d$. The Hamiltonians are given by:

(8) $${\hat{H}}_{A}={\hat{H}}_{B}=\sum_{k=0}^{d-1}\frac{2\pi }{T}k|k\rangle \langle k|$$

with

(9) $$E_{k}^{\left(A\right)}=E_{k}^{\left(B\right)}=\frac{2\pi }{T}k.$$

In introducing the time states of the clocks, we divide the discussion assuming clocks *A* and *B* with discrete or with continuous time values. In the first case, we have:

(10) $${|\tau_{m}\rangle }_{A}=\frac{1}{\sqrt{d}}\sum_{k=0}^{d-1}e^{-i\frac{2\pi }{T}k\tau_{m}}{|k\rangle }_{A}=\frac{1}{\sqrt{d}}\sum_{k=0}^{d-1}e^{-i\frac{2\pi }{d}km}{|k\rangle }_{A}$$

and

(11) $${|\theta_{l}\rangle }_{B}=\frac{1}{\sqrt{d}}\sum_{k=0}^{d-1}e^{-i\frac{2\pi }{T}k\theta_{l}}{|k\rangle }_{B}=\frac{1}{\sqrt{d}}\sum_{k=0}^{d-1}e^{-i\frac{2\pi }{d}kl}{|k\rangle }_{B}$$

where we have defined $\tau_{m}=m\frac{T}{d}$ and $\theta_{l}=l\frac{T}{d}$ (m,l=0,1,2,…,d−1). The time values are therefore uniformly spread over the range *T*. The states (10) and (11) satisfy $\langle \tau_{m^{\prime }}|\tau_{m}\rangle =\delta_{m,m^{\prime }}$ and $\langle \theta_{l^{\prime }}|\theta_{l}\rangle =\delta_{l,l^{\prime }}$. When considering clocks with continuous time values, we can instead introduce:

(12) $${|{\tilde{\tau }}_{f}\rangle }_{A}=\sum_{k=0}^{d-1}e^{-i\frac{2\pi }{T}k{\tilde{\tau }}_{f}}{|k\rangle }_{A}=\sum_{k=0}^{d-1}e^{-i2\pi kf}{|k\rangle }_{A}$$

and

(13) $${|{\tilde{\theta }}_{g}\rangle }_{B}=\sum_{k=0}^{d-1}e^{-i\frac{2\pi }{T}k{\tilde{\theta }}_{g}}{|k\rangle }_{B}=\sum_{k=0}^{d-1}e^{-i2\pi kg}{|k\rangle }_{B}$$

where we have defined ${\tilde{\tau }}_{f}=fT$ and ${\tilde{\theta }}_{g}=gT$, with *f* and *g* taking any real values in $\left[0,1\right]$.

### 2.3. Evolution of A+B

Let us now look at the heart of PaW’s theory. Through resolution (7), the condensed history of the system A+B can be written in the entangled global stationary state |Ψ〉, which satisfies the constraint (4). We want clocks *A* and *B* to be uncorrelated, so we assume them in a product state, thus obtaining:

(14) $$|\Psi \rangle =\frac{1}{T_{C}}\int_{0}^{T_{C}}dt{|t\rangle }_{C}⊗{|\varphi (t)\rangle }_{A}⊗{|\phi (t)\rangle }_{B}$$

where we have chosen as initial time $t_{0}=0$. In this framework, the relative state (in Everett’s sense [29]) of A+B with respect to *C* can be obtained via conditioning:

(15) $${|\varphi (t)\rangle }_{A}⊗{|\phi (t)\rangle }_{B}=\langle t|\Psi \rangle .$$

Note that, as mentioned before, Equation (15) is the Everett relative state definition of the subsystem *S* with respect to the subsystem *C*. As pointed out in [14], this kind of projection has nothing to do with a measurement. Rather, ${|\varphi (t)\rangle }_{A}⊗{|\phi (t)\rangle }_{B}$ is a state of A+B conditioned to having the state ${|t\rangle }_{C}$ in the subspace *C*.

For the initial state of the clocks, here, we choose:

(16) $${|\varphi (0)\rangle }_{A}⊗{|\phi (0)\rangle }_{B}\propto \sum_{k=0}^{d-1}{|k\rangle }_{A}⊗\sum_{n=0}^{d-1}{|n\rangle }_{B}$$

namely, we consider, at time t=0, the clocks *A* and *B* to be in the time states ${|\tau_{m=0}\rangle }_{A}$ and ${|\theta_{l=0}\rangle }_{B}$ (or ${|{\tilde{\tau }}_{f=0}\rangle }_{A}$ and ${|{\tilde{\theta }}_{g=0}\rangle }_{B}$ when considering continuous time values). Thus, from Equations (3), (4) and (15), it is possible to demonstrate that the state of A+B at generic time *t* reads [6]:

(17) $$\begin{array}{cc} {|\varphi (t)\rangle }_{A}⊗{|\phi (t)\rangle }_{B} & =e^{-i\left({\hat{H}}_{A}+{\hat{H}}_{B}\right)t}{|\varphi (0)\rangle }_{A}⊗{|\phi (0)\rangle }_{B}\\ & \propto \sum_{k=0}^{d-1}e^{-i\frac{2\pi }{T}kt}{|k\rangle }_{A}⊗\sum_{n=0}^{d-1}e^{-i\frac{2\pi }{T}nt}{|n\rangle }_{B} \end{array}$$

showing the Schrödinger evolution for the product state of A+B with respect to the time reference *C*. We can easily verify that *A* and *B* evolve synchronously. In the case of discrete time values, at time $t=m\frac{T}{d}$, we have:

(18) $${|\varphi (t=m\frac{T}{d})\rangle }_{A}⊗{|\phi (t=m\frac{T}{d})\rangle }_{B}\propto \sum_{k=0}^{d-1}e^{-i\frac{2\pi }{d}km}{|k\rangle }_{A}⊗\sum_{n=0}^{d-1}e^{-i\frac{2\pi }{d}nm}{|n\rangle }_{B}$$

where *A* reaches the state ${|\tau_{m}\rangle }_{A}$ and *B* is reaches the state ${|\theta_{l=m}\rangle }_{B}$. The same holds for clocks with continuous time values. Indeed, at time t=fT, we have:

(19) $${|\varphi (t=fT)\rangle }_{A}⊗{|\phi (t=fT)\rangle }_{B}\propto \sum_{k=0}^{d-1}e^{-i2\pi kf}{|k\rangle }_{A}⊗\sum_{n=0}^{d-1}e^{-i2\pi nf}{|n\rangle }_{B}.$$

Also in this case, we can see *A* and *B* simultaneously reach the states ${|{\tilde{\tau }}_{f}\rangle }_{A}$ and ${|{\tilde{\theta }}_{g=f}\rangle }_{B}$.

## 3. Evolution of Perturbed Clocks

**Figure 1:**
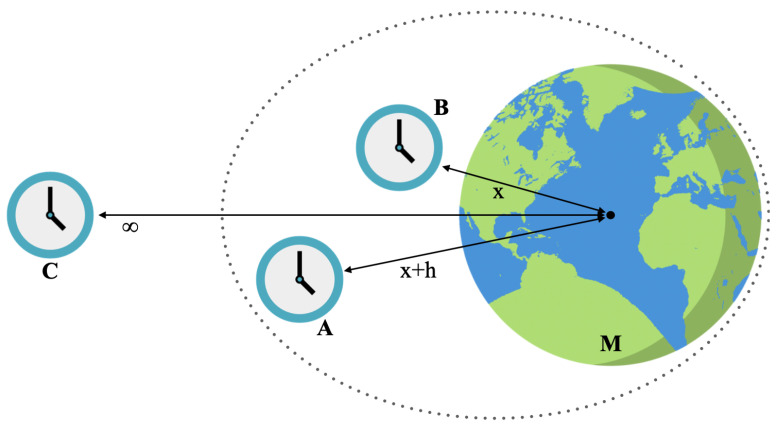
The clocks *A* and *B* are placed in the gravitational potential at distance x+h and *x*, respectively, from the center of the spherical mass *M*. Their evolution is studied (via the PaW formalism) with respect to the far-away observer *C*.

We consider now the case in which clocks *A* and *B* are placed in the gravitational field. We assume *B* at a distance *x* from the center of a spherical mass *M* and *A* placed at a distance x+h (see Figure 1). When considering the relativistic gravitational potential, the energy *V* of a clock placed at a distance *x* from the origin of the field reads [2,3]:

(20) $$V=m_{clock}\left[{\left(1-\frac{2GM}{x}\right)}^{\frac{1}{2}}-1\right].$$

As in [7,9,10], we treat the coordinate *x* as a number and, in calculating the gravitational interaction, we promote the masses to operators using the mass–energy equivalence: $m_{A}\to m_{A}+{\hat{H}}_{A}$ and $m_{B}\to m_{B}+{\hat{H}}_{B}$. Since the contributions given by the static masses would only lead to unobservable global phase factors in the evolution of the clocks, we do not consider them in the discussion. Furthermore, following [7], we assume the clocks to follow semiclassical trajectories which are approximately static, namely, with approximately zero velocity with respect to the mass *M* and the far-away observer *C*.

### 3.1. *A* and *B* in the Gravitational Field

The global Hamiltonian, including the interaction terms with the field, reads:

(21) $$\hat{H}={\hat{H}}_{C}+{\hat{H}}_{A}{\left(1-\frac{2GM}{x+h}\right)}^{\frac{1}{2}}+{\hat{H}}_{B}{\left(1-\frac{2GM}{x}\right)}^{\frac{1}{2}}={\hat{H}}_{C}+{\hat{H^{\prime }}}_{A}+{\hat{H^{\prime }}}_{B}$$

where now,

(22) $${\hat{H^{\prime }}}_{A}={\hat{H}}_{A}{\left(1-\frac{2GM}{x+h}\right)}^{\frac{1}{2}}=\sum_{k=0}^{d-1}\frac{2\pi }{T^{\prime \prime }}k|k\rangle \langle k|$$

and

(23) $${\hat{H^{\prime }}}_{B}={\hat{H}}_{B}{\left(1-\frac{2GM}{x}\right)}^{\frac{1}{2}}=\sum_{k=0}^{d-1}\frac{2\pi }{T^{\prime }}k|k\rangle \langle k|$$

with $T^{\prime \prime }=T{\left(1-\frac{2GM}{x+h}\right)}^{-\frac{1}{2}}$ and $T^{\prime }=T{\left(1-\frac{2GM}{x}\right)}^{-\frac{1}{2}}$.

We introduce again the time states. In the case of clocks with discrete time values, we have:

(24) $${|\tau_{m}\rangle }_{A}=\frac{1}{\sqrt{d}}\sum_{k=0}^{d-1}e^{-i\frac{2\pi }{T^{\prime \prime }}k\tau_{m}}{|k\rangle }_{A}=\frac{1}{\sqrt{d}}\sum_{k=0}^{d-1}e^{-i\frac{2\pi }{d}km}{|k\rangle }_{A}$$

and

(25) $${|\theta_{l}\rangle }_{B}=\frac{1}{\sqrt{d}}\sum_{k=0}^{d-1}e^{-i\frac{2\pi }{T^{\prime }}k\theta_{l}}{|k\rangle }_{B}=\frac{1}{\sqrt{d}}\sum_{k=0}^{d-1}e^{-i\frac{2\pi }{d}kl}{|k\rangle }_{B}$$

where we have redefined $\tau_{m}=m\frac{T^{\prime \prime }}{d}=m\frac{T}{d{\left(1-\frac{2GM}{x+h}\right)}^{\frac{1}{2}}}$ and $\theta_{l}=l\frac{T^{\prime }}{d}=l\frac{T}{d{\left(1-\frac{2GM}{x}\right)}^{\frac{1}{2}}}$. We notice and emphasize that the presence of the gravitational field does not change the form of the time states. The same holds for the case of clocks with continuous time values. Indeed, in this latter case, we have:

(26) $${|{\tilde{\tau }}_{f}\rangle }_{A}=\sum_{k=0}^{d-1}e^{-i\frac{2\pi }{T^{\prime \prime }}k{\tilde{\tau }}_{f}}{|k\rangle }_{A}=\sum_{k=0}^{d-1}e^{-i2\pi kf}{|k\rangle }_{A}$$

and

(27) $${|{\tilde{\theta }}_{g}\rangle }_{B}=\sum_{k=0}^{d-1}e^{-i\frac{2\pi }{T^{\prime }}k{\tilde{\theta }}_{g}}{|k\rangle }_{B}=\sum_{k=0}^{d-1}e^{-i2\pi kg}{|k\rangle }_{B}$$

where now, ${\tilde{\tau }}_{f}=fT^{\prime \prime }$ and ${\tilde{\theta }}_{g}=gT^{\prime }$ with $f,g\in \left[0,1\right]$.

We investigate the time evolution of A+B in this new scenario. The global state satisfying the global constraint (4) can again be written as in (14), and we also assume here the clocks are starting in the product state (16). When the observer *C* reads the generic time *t*, we have:

(28) $${|\varphi (t)\rangle }_{A}⊗{|\phi (t)\rangle }_{B}\propto \sum_{k=0}^{d-1}e^{-i\frac{2\pi }{T}kt{\left(1-\frac{2GM}{x+h}\right)}^{\frac{1}{2}}}{|k\rangle }_{A}⊗\sum_{n=0}^{d-1}e^{-i\frac{2\pi }{T}nt{\left(1-\frac{2GM}{x}\right)}^{\frac{1}{2}}}{|n\rangle }_{B}.$$

In the case of clocks with discrete time values, considered to be at time $t=m\frac{T}{d}$, Equation (28) becomes:

(29) $${|\varphi (t=m\frac{T}{d})\rangle }_{A}⊗{|\phi (t=m\frac{T}{d})\rangle }_{B}\propto \sum_{k=0}^{d-1}e^{-i\frac{2\pi }{d}km{\left(1-\frac{2GM}{x+h}\right)}^{\frac{1}{2}}}{|k\rangle }_{A}⊗\sum_{n=0}^{d-1}e^{-i\frac{2\pi }{d}nm{\left(1-\frac{2GM}{x}\right)}^{\frac{1}{2}}}{|n\rangle }_{B}.$$

This implies that when *A* reaches the time state $|\tau_{m^{\prime \prime }}\rangle$, clock *B* has reached a number of states:

(30) $$m^{\prime }=m^{\prime \prime }{\left(1-\frac{2GM}{x}\right)}^{\frac{1}{2}}{\left(1-\frac{2GM}{x+h}\right)}^{-\frac{1}{2}}$$

which is in agreement with the time dilation between two clocks at a (radial) distance *h* from each other, as obtained from the Schwarzschild solution (see Equation (2)).

Similarly, when considering clocks with continuous time values, assumed to be at t=fT, Equation (28) becomes:

(31) $${|\varphi (t=fT)\rangle }_{A}⊗{|\phi (t=fT)\rangle }_{B}\propto \sum_{k=0}^{d-1}e^{-i2\pi kf{\left(1-\frac{2GM}{\left(x+h\right)c^{2}}\right)}^{\frac{1}{2}}}{|k\rangle }_{A}⊗\sum_{n=0}^{d-1}e^{-i2\pi nf{\left(1-\frac{2GM}{xc^{2}}\right)}^{\frac{1}{2}}}{|n\rangle }_{B}.$$

This implies that when *A* reaches the time state ${|{\tilde{\tau }}_{f^{\prime \prime }}\rangle }_{A}$, the clock *B* reaches the time state ${|{\tilde{\theta }}_{g=f^{\prime }}\rangle }_{B}$ with:

(32) $$f^{\prime }=f^{\prime \prime }{\left(1-\frac{2GM}{x}\right)}^{\frac{1}{2}}{\left(1-\frac{2GM}{x+h}\right)}^{-\frac{1}{2}}.$$

We can also put clock *A* at an infinite distance from the mass. By taking h⟶∞, Equations (30) and (32) become $m^{\prime }=m^{\prime \prime }{\left(1-\frac{2GM}{x}\right)}^{\frac{1}{2}}$ and $f^{\prime }=f^{\prime \prime }{\left(1-\frac{2GM}{x}\right)}^{\frac{1}{2}}$, again, in agreement with the temporal term in the Schwarzschild metric

(33) $$d\tau ={\left(1-\frac{2GM}{x}\right)}^{\frac{1}{2}}dt$$

where dτ is the infinitesimal proper time read within the field, and dt is the coordinate time that can be considered as read by a far-away observer.

### 3.2. Introducing the Proper Time

In this paragraph, we briefly reinterpret what we have just discussed by introducing the proper time read by the clocks *A* and *B*. In Equation (28), we found that when the observer *C* read the generic time *t*, the states of A+B evolved according to:

(34) $${|\varphi (t)\rangle }_{A}⊗{|\phi (t)\rangle }_{B}\propto \sum_{k=0}^{d-1}e^{-i\frac{2\pi }{T}kt{\left(1-\frac{2GM}{x+h}\right)}^{\frac{1}{2}}}{|k\rangle }_{A}⊗\sum_{n=0}^{d-1}e^{-i\frac{2\pi }{T}nt{\left(1-\frac{2GM}{x}\right)}^{\frac{1}{2}}}{|n\rangle }_{B}.$$

We can now define the proper times read by clocks *A* and *B* as $\tau_{A}\left(t\right)=t{\left(1-\frac{2GM}{x+h}\right)}^{\frac{1}{2}}$ and $\tau_{B}\left(t\right)=t{\left(1-\frac{2GM}{x}\right)}^{\frac{1}{2}}$. Equation (34) can thus be rewritten as:

(35) $${|\varphi (t)\rangle }_{A}⊗{|\phi (t)\rangle }_{B}\propto \sum_{k=0}^{d-1}e^{-i\frac{2\pi }{T}k\tau_{A}\left(t\right)}{|k\rangle }_{A}⊗\sum_{n=0}^{d-1}e^{-i\frac{2\pi }{T}n\tau_{B}\left(t\right)}{|n\rangle }_{B}$$

where it is manifest that in the product state of A+B, each clock evolves according to its proper time.

This result can also be summarized by writing:

(36) $$\begin{array}{cc} {|\varphi (t)\rangle }_{A}⊗{|\phi (t)\rangle }_{B} & =e^{-i\;\left({\hat{H^{\prime }}}_{A}+{\hat{H^{\prime }}}_{B}\right)t}{|\varphi (0)\rangle }_{A}⊗{|\phi (0)\rangle }_{B}\\ & =e^{-i{\hat{H}}_{A}\tau_{A}\left(t\right)}{|\varphi (0)\rangle }_{A}⊗e^{-i{\hat{H}}_{B}\tau_{B}\left(t\right)}{|\phi (0)\rangle }_{B} \end{array}$$

clearly showing that the effect of interaction with the gravitational potential can be interpreted as (properly) dilating time, while leaving the clocks energy unchanged.

## 4. Evolution of Entangled Clocks

In this section, we study the evolution of clocks *A* and *B* in a generic (i.e., not product) state. We consider the clocks in the gravitational potential, with *B* at a distance *x* from the center of the mass *M*, and *A* at a distance x+h, as in the previous section. The global state satisfying the global constraint (4) can be written as:

(37) $$\begin{array}{cc} |\Psi \rangle & =\frac{1}{T_{C}}\int_{0}^{T_{C}}dt{|t\rangle }_{C}⊗{|\psi (t)\rangle }_{AB}\\ & =\frac{1}{T_{C}}\int_{0}^{T_{C}}dt{|t\rangle }_{C}⊗e^{-i{\hat{H}}_{AB}t}{|\psi (0)\rangle }_{AB} \end{array}$$

where ${\hat{H}}_{AB}$ and ${|\psi (0)\rangle }_{AB}$ are the Hamiltonian and the initial state referring to the subsystem A+B of the clocks. We start by exploring the dynamics of the clocks in this new case, and then we consider the case in which an interaction term between the clocks is present.

### 4.1. Clocks *A* and *B* in a Generic State

We take the initial state of A+B appearing in (37) as the generic state in the energy eigenbasis:

(38) $${|\psi (0)\rangle }_{AB}=\sum_{n=0}^{d-1}\sum_{k=0}^{d-1}c_{nk}{|n\rangle }_{A}⊗{|k\rangle }_{B}$$

where $\sum_{n,k}{\left|c_{nk}\right|}^{2}=1$, and we calculate its evolution through the Hamiltonian

(39) $${\hat{H}}_{AB}={\hat{H}}_{A}{\left(1-\frac{2GM}{x+h}\right)}^{\frac{1}{2}}+{\hat{H}}_{B}{\left(1-\frac{2GM}{x}\right)}^{\frac{1}{2}}.$$

Using the results of Section 3.2, we obtain:

(40) $$\begin{array}{cc} {|\psi (t)\rangle }_{AB} & =e^{-i{\hat{H}}_{AB}t}{|\psi (0)\rangle }_{AB}\\ & =e^{-i\left({\hat{H}}_{A}{\left(1-\frac{2GM}{x+h}\right)}^{\frac{1}{2}}+{\hat{H}}_{B}{\left(1-\frac{2GM}{x}\right)}^{\frac{1}{2}}\right)t}{|\psi (0)\rangle }_{AB}\\ & =e^{-i{\hat{H}}_{A}\tau_{A}\left(t\right)}e^{-i{\hat{H}}_{B}\tau_{B}\left(t\right)}{|\psi (0)\rangle }_{AB}\\ & =\sum_{n=0}^{d-1}\sum_{k=0}^{d-1}c_{nk}e^{-i\frac{2\pi }{T}n\tau_{A}\left(t\right)}{|n\rangle }_{A}⊗e^{-i\frac{2\pi }{T}k\tau_{B}\left(t\right)}{|k\rangle }_{B}\\ & =\sum_{n=0}^{d-1}\sum_{k=0}^{d-1}c_{nk}e^{-i\frac{2\pi }{T}\left(n\tau_{A}\left(t\right)+k\tau_{B}\left(t\right)\right)}{|n\rangle }_{A}⊗{|k\rangle }_{B} \end{array}$$

where $\tau_{A}\left(t\right)$ and $\tau_{B}\left(t\right)$ are the proper times read by clocks *A* and *B*, respectively, when the observer in *C* reads time *t*. From Equation (40), we can easily see how within a generic state of A+B, each term in the superposition acquires a phase proportional to the sum of integer multiples of proper times read by the two clocks.

To better understand this kind of evolution, we can look at a simple example by taking *d* = 2. Namely, we consider, for *A* and *B*, the simplest choice of clock: a qubit. In Section 2 and Section 3, we assumed the ground state of the Hamiltonian with zero energy, but the framework can be easily generalized for energy translations. Thus, we take:

(41) $${\hat{H}}_{A}={\hat{H}}_{B}=\frac{\omega }{2}{\hat{\sigma }}_{z}$$

leading to the Hamiltonian:

(42) $${\hat{H}}_{AB}=\frac{\omega }{2}{\hat{\sigma }}_{z}^{\left(A\right)}{\left(1-\frac{2GM}{x+h}\right)}^{\frac{1}{2}}+\frac{\omega }{2}{\hat{\sigma }}_{z}^{\left(B\right)}{\left(1-\frac{2GM}{x}\right)}^{\frac{1}{2}}.$$

The (initial) generic state of two qubits can be written as:

(43) $${|\psi (0)\rangle }_{AB}=\alpha |00\rangle +\beta |01\rangle +\gamma |10\rangle +\delta |11\rangle$$

with ${\left|\alpha \right|}^{2}+{\left|\beta \right|}^{2}+{\left|\gamma \right|}^{2}+{\left|\delta \right|}^{2}=1$. In the state (43), the first position in the kets refers to clock *A* and the second to clock *B*. We can now write:

(44) $$\begin{array}{c} e^{-i\frac{\omega }{2}{\hat{\sigma }}_{z}^{\left(A\right)}{\left(1-\frac{2GM}{x+h}\right)}^{\frac{1}{2}}t}=\left(\begin{array}{cc} e^{-i\frac{\omega }{2}\tau_{A}\left(t\right)} & 0\\ 0 & e^{i\frac{\omega }{2}\tau_{A}\left(t\right)} \end{array}\right) \end{array}$$

and similarly,

(45) $$\begin{array}{c} e^{-i\frac{\omega }{2}{\hat{\sigma }}_{z}^{\left(B\right)}{\left(1-\frac{2GM}{x+h}\right)}^{\frac{1}{2}}t}=\left(\begin{array}{cc} e^{-i\frac{\omega }{2}\tau_{B}\left(t\right)} & 0\\ 0 & e^{i\frac{\omega }{2}\tau_{B}\left(t\right)} \end{array}\right). \end{array}$$

The time evolution of (43) can be easily calculated:

(46) $$\begin{array}{c} {|\psi (t)\rangle }_{AB}=\alpha e^{-i\frac{\omega }{2}\left(\tau_{A}\left(t\right)+\tau_{B}\left(t\right)\right)}|00\rangle +\beta e^{-i\frac{\omega }{2}\left(\tau_{A}\left(t\right)-\tau_{B}\left(t\right)\right)}|01\rangle +\\ +\gamma e^{i\frac{\omega }{2}\left(\tau_{A}\left(t\right)-\tau_{B}\left(t\right)\right)}|10\rangle +\delta e^{i\frac{\omega }{2}\left(\tau_{A}\left(t\right)+\tau_{B}\left(t\right)\right)}|11\rangle \end{array}$$

where it is manifest that the phases acquired by the various states in the superposition depend on sums or differences (remember that here, the energy spectrum of *A* and *B* has values $\pm \frac{\omega }{2}$) of the two clocks’ proper times.

Finally, we rewrite (46) as

(47) $${|\psi (t)\rangle }_{AB}=\alpha \left(t\right)|00\rangle +\beta \left(t\right)|01\rangle +\gamma \left(t\right)|10\rangle +\delta \left(t\right)|11\rangle$$

and we calculate the concurrence C(ψ(t))=2|α(t)δ(t)−β(t)γ(t)|, to keep track of the measure of entanglement over time [30,31]. We find:

(48) $$\begin{array}{cc} C(\psi (t)) & =2\left|\alpha (t)\delta (t)-\beta (t)\gamma (t)\right|\\ & =2|\alpha e^{-i\frac{\omega }{2}\left(\tau_{A}\left(t\right)+\tau_{B}\left(t\right)\right)}\delta e^{i\frac{\omega }{2}\left(\tau_{A}\left(t\right)+\tau_{B}\left(t\right)\right)}-\beta e^{-i\frac{\omega }{2}\left(\tau_{A}\left(t\right)-\tau_{B}\left(t\right)\right)}\gamma e^{i\frac{\omega }{2}\left(\tau_{A}\left(t\right)-\tau_{B}\left(t\right)\right)}|\\ & =2\left|\alpha \delta -\beta \gamma \right|=C\left(\psi \left(0\right)\right) \end{array}$$

showing (as expected) that the interaction with the gravitational potential is not able to change the measure of the entanglement present in the initial state.

### 4.2. Entanglement of Interacting Clocks

In order to observe a change in the measure of entanglement during the clocks’ dynamics, it is necessary to introduce a term of interaction between them. For this reason, in this paragraph, we study the time evolution of *A* and *B* described by two qubits with the addition of a simple interaction term ${\hat{H}}_{int}=\epsilon {\hat{H}}_{A}⊗{\hat{H}}_{B}$ in the Hamiltonian ${\hat{H}}_{AB}$. This form of interaction is obtained, for example, if one considers the clocks interacting through Newtonian gravity, by taking ϵ=−G/h with *h* the distance between *A* and *B*.

The initial state of clocks *A* and *B* is again given by (43), and we calculate its evolution through the Hamiltonian:

(49) $${\hat{H}}_{AB}=\frac{\omega }{2}{\hat{\sigma }}_{z}^{\left(A\right)}{\left(1-\frac{2GM}{x+h}\right)}^{\frac{1}{2}}+\frac{\omega }{2}{\hat{\sigma }}_{z}^{\left(B\right)}{\left(1-\frac{2GM}{x}\right)}^{\frac{1}{2}}+\frac{\epsilon \omega^{2}}{2}{\hat{\sigma }}_{z}^{\left(A\right)}⊗{\hat{\sigma }}_{z}^{\left(B\right)}.$$

Thus, the state of A+B at generic time *t* reads:

(50) $${|\psi (t)\rangle }_{AB}=e^{-i\frac{\omega }{2}\tau_{A}\left(t\right){\hat{\sigma }}_{z}^{\left(A\right)}}e^{-i\frac{\omega }{2}\tau_{B}\left(t\right){\hat{\sigma }}_{z}^{\left(B\right)}}e^{-i\frac{\epsilon \omega^{2}}{2}t{\hat{\sigma }}_{z}^{\left(A\right)}⊗{\hat{\sigma }}_{z}^{\left(B\right)}}{|\psi (0)\rangle }_{AB}$$

which leads to the result

(51) $$\begin{array}{c} {|\psi (t)\rangle }_{AB}=\alpha e^{-i\frac{\omega }{2}\left(\tau_{A}\left(t\right)+\tau_{B}\left(t\right)\right)}e^{-i\frac{\epsilon \omega^{2}}{2}t}|00\rangle +\beta e^{-i\frac{\omega }{2}\left(\tau_{A}\left(t\right)-\tau_{B}\left(t\right)\right)}e^{i\frac{\epsilon \omega^{2}}{2}t}|01\rangle +\\ +\gamma e^{i\frac{\omega }{2}\left(\tau_{A}\left(t\right)-\tau_{B}\left(t\right)\right)}e^{i\frac{\epsilon \omega^{2}}{2}t}|10\rangle +\delta e^{i\frac{\omega }{2}\left(\tau_{A}\left(t\right)+\tau_{B}\left(t\right)\right)}e^{-i\frac{\epsilon \omega^{2}}{2}t}|11\rangle \end{array}$$

where we can see that, in addition to the phases containing the proper times of the clocks, the time *t* read by the observer *C* also explicitly appears.

We can now again rewrite Equation (51) as:

(52) $${|\psi (t)\rangle }_{AB}=\alpha \left(t\right)|00\rangle +\beta \left(t\right)|01\rangle +\gamma \left(t\right)|10\rangle +\delta \left(t\right)|11\rangle$$

and calculate the concurrence C(ψ(t))=2|α(t)δ(t)−β(t)γ(t)|. We obtain:

(53) $$C\left(\psi \left(t\right)\right)=2\left|\alpha \delta e^{-i\epsilon \omega^{2}t}-\beta \gamma e^{i\epsilon \omega^{2}t}\right|$$

where we can immediately observe that the dependence on proper times vanishes, while the contributions associated with time *t* persist. Equation (53) thus shows that the concurrence is in general oscillating with time *t*. If, for example, we choose $\alpha \delta =\beta \gamma =\frac{1}{4}$, we obtain $C\left(\psi \left(t\right)\right)=|\sin\left(\epsilon \omega^{2}t\right)|$, displaying an initially unentangled state, whose concurrence subsequently oscillates between zero and nonzero values as time *t* evolves.

We observe that, given the form of our interaction, it is sufficient for one of the coefficients α,β,γ and δ to be equal to zero in order for the concurrence to remain constant in time. An example is provided by the Bell states, for which we have $\alpha \delta =\pm \frac{1}{2},\beta \gamma =0$ or $\alpha \delta =0,\beta \gamma =\pm \frac{1}{2}$. In such cases, it is straightforward to show that the entanglement remains maximal at all times, with C(ψ(t))=1.

Finally, we note that gravitationally induced entanglement between clocks, based on the mass–energy equivalence principle, was already introduced and studied in [7]. In that work, the authors focused on decoherence and the breakdown of time measurability caused by gravitational interactions between quantum clocks. Our aim, by contrast, was to investigate how the entanglement between interacting clocks transformed when described from the perspective of a reference clock *C*, within the PaW framework. A similar type of interaction was also assumed in [9], where the PaW formalism was actually considered. However, in this latter work, the authors focused on the interaction between the reference clock and the rest of the universe. In that setting, they derived a modified Schrödinger equation for the rest, resulting from the interaction with the reference clock. In summary, while previous works focused on gravitational effects on time measurability or dynamics, our contribution was to examine how the entanglement between two interacting clocks (within a gravitational field) appeared when studied in a fully relational description.

## 5. Gravitational Redshift

We derive here the gravitational redshift as emerging in our framework. For this section, we introduce c≠1.

We consider *A* and *B* both placed in the gravitational potential at distance x+h and *x*, respectively, from the origin of the field (the Hamiltonians are given by (22) and (23)), and we assume an observer in *A* receiving a light signal emitted in *B*. We assume the frequency of the light signal as proportional to the spacing between two neighboring energy levels of the clocks, namely, 1/T for a free clock. The observer *A* can thus read the frequency $\nu_{O}$ coming from *B* and compare it with their own spectrum, that is, $\delta \nu =\nu_{O}-\nu =\frac{1}{T^{\prime }}-\frac{1}{T^{\prime \prime }}$, leading to

(54) $$\delta \nu =\frac{1}{T}\left[{\left(1-\frac{2GM}{xc^{2}}\right)}^{\frac{1}{2}}-{\left(1-\frac{2GM}{\left(x+h\right)c^{2}}\right)}^{\frac{1}{2}}\right].$$

At the first order of approximation, when $\frac{2GM}{xc^{2}}\ll 1$, we therefore have:

(55) $$\delta \nu ≃\frac{1}{T}\left[\left(1-\frac{GM}{xc^{2}}\right)-\left(1-\frac{GM}{\left(x+h\right)c^{2}}\right)\right]$$

which, for h≪x, becomes

(56) $$\delta \nu ≃\frac{1}{T}\frac{GM}{c^{2}}\left(\frac{1}{x+h}-\frac{1}{x}\right)≃-\frac{1}{T}\frac{GMh}{x^{2}c^{2}}.$$

Writing now the gravitational acceleration $a=\frac{GM}{x^{2}}$ and neglecting terms of the order ∼${\left(\frac{GM}{xc^{2}}\right)}^{2}$, we obtain

(57) $$\frac{\delta \nu }{\nu }≃-\frac{ah}{c^{2}}.$$

Equation (57) is in agreement with what has been measured in experiments on Earth (see, for example, Ref. [32]). It clearly holds when considering the spacing between any two energy levels and not only between two neighbors.

## 6. Conclusions

In this work, through the PaW theory, we examined the time evolution of two quantum clocks (*A* and *B*) when interacting with a relativistic gravitational potential. We conducted our investigation when clocks had discrete and continuous time values. In both cases, we first verified that in the absence of the field, the two clocks evolved synchronously. Then, promoting the mass of the clocks to operator, we introduced the interaction with the field, and we found a time dilation effect for the time states of the clocks in agreement with the Schwarzschild solution (1). By introducing the proper time for the clocks, we thus showed that the effect of the interaction with the gravitational potential could be interpreted as (properly) dilating time, while leaving the clocks energy unchanged. The evolution of entangled clocks was also studied, and the expression for the gravitational redshift was derived and discussed.

As subject of future work, we propose to also introduce the spatial degree of freedom in the discussion. Through the interaction of a quantum ruler with the relativistic gravitational potential, we will be able (hopefully) to show that the possible outcomes of a position measurement made on the ruler placed in the gravitational field are modified in agreement with the gravitational lengths stretching, as obtained from the Schwarzschild metric. Through the study of a time-evolving ruler, we thus would derive and discuss the probability that the ruler connects events in spacetime. We notice that this proposal would be carried out in a fully relational approach, moving the discussion into the more general context of quantum reference frames [15,26].

Finally, we emphasize that the choice to use the distance *x* from the origin of the field as a number is only an approximation, useful to show the power of the framework; it cannot be the ultimate solution. We thus propose to move away from this approximation in the future by providing a framework where the distance to the clocks from the origin of the field is treated as an operator.

## Data Availability

No new data were produced in this study.
